# Early radiologic signal of responsiveness to immune checkpoint blockade in microsatellite-stable/mismatch repair-proficient metastatic colorectal cancer

**DOI:** 10.1038/s41416-022-02004-0

**Published:** 2022-10-13

**Authors:** Sebastian Meltzer, Anne Negård, Kine M. Bakke, Hanne M. Hamre, Christian Kersten, Eva Hofsli, Marianne G. Guren, Halfdan Sorbye, Kjersti Flatmark, Anne Hansen Ree

**Affiliations:** 1 Department of Oncology, Akershus University Hospital, Lørenskog, Norway; 2 Department of Radiology, Akershus University Hospital, Lørenskog, Norway; 3 Institute of Clinical Medicine, University of Oslo, Oslo, Norway; 4 Department of Research, Sørlandet Hospital, Kristiansand, Norway; 5 Department of Oncology, St. Olav’s Hospital, Trondheim, Norway; 6 Department of Clinical and Molecular Medicine, Norwegian University of Science and Technology, Trondheim, Norway; 7 Department of Oncology, Oslo University Hospital, Oslo, Norway; 8 Department of Oncology, Haukeland University Hospital, Bergen, Norway; 9 Department of Clinical Science, University of Bergen, Bergen, Norway; 10 Department of Gastroenterological Surgery, Oslo University Hospital, Oslo, Norway; 11 Department of Tumour Biology, Oslo University Hospital, Oslo, Norway

**Keywords:** Colorectal cancer, Prognostic markers, Cancer imaging, Prognostic markers

## Abstract

**Background:**

Immune checkpoint blockade (ICB) results in radiologic tumour response dynamics that differ from chemotherapy efficacy measures and require an early signal of clinical utility.

**Methods:**

Previously untreated, unresectable microsatellite-stable (MSS)/mismatch repair-proficient (pMMR) colorectal cancer (CRC) patients were randomly assigned to the oxaliplatin-based Nordic FLOX regimen (control arm) or repeat sequential two FLOX cycles and two ICB cycles (experimental arm). The radiologic response was assessed every 8 weeks. In this *post hoc* analysis, we explored early target lesion (TL) dynamics as indicator of ICB responsiveness. Progression-free survival (PFS) was the primary endpoint.

**Results:**

Using a landmark analysis approach, we categorised experimental-arm patients into ≥10% (*N* = 19) or <10% (*N* = 16) TL reduction at the first post-baseline response assessment. Median PFS for the groups was 16.0 (95% confidence interval (CI), 12.3–19.7) and 3.9 months (95% CI, 2.3–5.5), respectively, superior and inferior (both *P* < 0.01) to the median PFS of 9.8 months (95% CI, 4.9–14.7) for control arm patients (*N* = 31).

**Conclusions:**

Radiologic TL reduction of ≥10% at the first post-baseline response assessment identified patients with ICB-responsive metastatic MSS/pMMR-CRC. This pragmatic measure may be used to monitor patients in investigational ICB schedules, enabling early treatment adaptation for unresponsive cases.

**Trial registration:**

ClinicalTrials.gov number, NCT03388190 (02/01/2018).

## Background

The cytotoxic mode of action of chemotherapeutic agents often translates into measurable tumour shrinkage at an early radiologic assessment, guiding the selection of patients to the continuation of the therapy or the conclusion of treatment failure. The antitumour activity of immune checkpoint blockade (ICB), however, may result in tumour response patterns that differ from efficacy measures of direct cytotoxicity [1, 2]. Early in the immuno-oncology era, it was acknowledged that efficacious ICB therapies may lead to the initial increase in tumour size because of immune cell infiltration, delay in onset of measurable effects and possibly undulating radiologic responses under ongoing clinical activity [3]. The opposite case—primary ICB resistance—is also a concern, illustrated by the significant percentage of patients with metastatic microsatellite-instable (MSI)/mismatch repair (MMR)-deficient (dMMR) colorectal cancer (CRC) who experienced immediate progression on first-line pembrolizumab treatment [4], although a transient progression [5] or misdiagnosed microsatellite-stable (MSS)/MMR-proficient (pMMR) cases [6] might explain some ICB failures. Finally, with the increasing introduction of combined-modality treatments that include ICB, it is reasonable to expect further complexity of clinical activity patterns and challenges in interpreting response data. Of particular importance, the selection of patients for highly experimental ICB schedules, for instance in metastatic MSS/pMMR-CRC, calls for applicable and reliable signals of the activity or early failure that enable treatment adaptation and maintain patient safety.

CRC is a heterogeneous disease of high molecular complexity, which has individualised treatment based on biological characteristics [7]. Still, unresectable metastases, particularly in abdominal organs, remain the cause of severe morbidity and dismal survival [8]. Among ICB-treated patients in the small CRC subgroup of metastatic MSI/dMMR disease, 70–80% had an ongoing response at 24 months in first-line studies [4, 9]. However, the majority of metastatic CRC patients have MSS/pMMR disease that causes low tumour antigenicity [10] and thus, is largely unresponsive to current ICB strategies [11]. Retrospective analysis indicated that in a patient cohort of metastatic MSS/pMMR-CRC given ICB therapies, the presence of liver metastases was the most significant variable associated with rapid disease progression [12], potentially reflecting the de novo ICB resistance incited by the elimination of cytotoxic T cells from experimental liver metastasis models and the absence of this specific immune cell population in liver metastasis specimens from MSS/pMMR-CRC patients [13, 14].

Yet, preclinical [15, 16] and clinical evidence, including our own from studies applying short-course oxaliplatin-based chemotherapy in combined-modality treatment schedules [17–22], supports the notion that oxaliplatin may induce immunogenic cell death [23] in CRC and invoke efficacious antitumour immunity. This led us to hypothesise that the metastatic MSS/pMMR-CRC entity can be transformed into an immunogenic condition by two cycles of the oxaliplatin-containing Nordic FLOX regimen [24], and patients with previously untreated, the unresectable disease that predominantly comprises infradiaphragmatic manifestations may achieve therapeutic efficacy from the sequential addition of ICB therapy. In the still ongoing METIMMOX trial, we acknowledge that this treatment strategy is complex and highly exploratory, which has led us to search for an early signal of clinical activity or failure. Unconventional tumour response patterns in ICB studies have revealed that currently used imaging criteria may need further clarification to capture the clinical benefit of ICB [2, 25].

## Methods

### The clinical study

The study was approved by an independent ethics committee, the institutional review boards and the Norwegian Medicines Agency and conducted in accordance with the Declaration of Helsinki. Written informed consent was required for study participation. This was a multicentre, open-label, Phase 2 randomised controlled trial for patients with previously untreated, unresectable metastatic colorectal MSS/pMMR adenocarcinoma (also comprising the mucinous adenocarcinoma and signet-ring cell carcinoma entities). Tumour MSS/MMR status was determined at the local centres by routine PCR or immunohistochemistry analysis. Other essential inclusion criteria were infradiaphragmatic metastatic manifestation(s), Eastern Cooperative Oncology Group performance status 0–1, and serum C-reactive protein less than 60 mg/L. A period of less than 6 months since the discontinuation of adjuvant oxaliplatin-containing chemotherapy was an exclusion criterion. The study treatment was assigned by computerised central randomisation in a 1:1 ratio following the determination of *RAS/BRAF* mutational status (wild-type or any mutation) and primary tumour sidedness (right or left) for balanced distribution of these parameters between the study arms. Patients received either eight cycles of the oxaliplatin-based Nordic FLOX regimen Q2W (oxaliplatin 85 mg/m^2^ on day 1 and bolus 5-fluorouracil 500 mg/m^2^ and folinic acid 100 mg on days 1 and 2; control arm) or two cycles of FLOX Q2W before two cycles of nivolumab (240 mg flat dose) Q2W in a repeat sequential schedule to a total of eight cycles (experimental study arm), in both cases before treatment break until disease progression and reintroduction of a new treatment sequence (Supplementary Fig. S1) and with dosing delay and resumption criteria reflecting protocol-specified adverse events. The go-and-stop schedule (alternating active therapy and treatment break) was continued until the first confirmed disease progression on active therapy (defining the primary endpoint of progression-free survival; PFS), intolerable toxicity, withdrawal of consent or death (a PFS event), whichever occurred first.

### Radiologic procedures

Patients had baseline radiologic review (by computed tomography; CT) within 4 weeks of the start of treatment. Radiologic response assessment, undertaken every 8 weeks on active therapy and in break periods, was performed by blinded independent central review and according to RECIST v1.1 [26] as the primary method and iRECIST [27] as the subsidiary method. Here, measurable target lesions (TLs) were initially selected for review at each evaluation, and PFS was determined by the established scoring criteria for tumour responses (including responses of non-measurable non-target lesions and recording and measuring of any new lesions).

### The *post hoc* biomarker assay

**Fig. 1:**
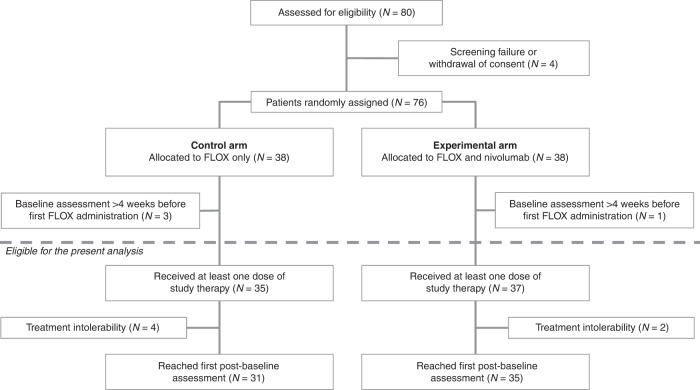
Flow diagram of the selection of cases for the current study. Out of 80 screened patients, 76 were randomised to receive either eight cycles of the Nordic FLOX regimen Q2W (control arm) or two cycles of FLOX Q2W followed by two cycles of nivolumab Q2W in a repeat sequential schedule to a total of eight cycles (experimental study arm). 31 patients in the control arm and 35 patients in the experimental study arm reached the first response assessment after 8 weeks of treatment.

PFS was the primary endpoint of this randomised controlled trial, and the results will be reported separately. Here, we have explored early markers of treatment response. Using a landmark analysis approach (to avoid bias against patients who left the study before the response could be formally evaluated) [28], patients with available response assessment at a minimum of 8 weeks were categorised according to the TL change at the first post-baseline assessment (Fig. 1). Median TL reduction at this earliest possible opportunity for treatment evaluation was 10.8% in the experimental arm (*N* = 35) and 26.7% in the control arm (*N* = 31). Thus, the group of experimental-arm patients was divided according to categories of ≥10% (*N* = 19) or <10% (*N* = 16) TL reduction at 8 weeks as a pragmatic cut-off. The control arm was divided similarly for comparison (≥10%, *N* = 25; <10%, *N* = 6).

### Magnetic resonance imaging (MRI) of liver metastases

The exploratory biomarker programme of the study comprised MRI acquisitions for examination of the dynamics of potential therapy responses in liver metastases. For some study patients at one centre, MRI of the liver was recorded at baseline, following the initial two FLOX cycles and then after the sequential two nivolumab cycles, irrespective of any dosing delays. This enabled alternative-modality assessment of TL changes and calculation of changes in volume and apparent diffusion coefficient (ADC) of liver metastases. High-resolution axial T2-weighted images were used to manually draw metastatic regions, from which the tumour volume was estimated. A diffusion-weighted sequence with six *b*-values (0, 25, 50, 100, 500 and 1000 s/mm^2^) was used to estimate ADC values with a standard linear fit approach.

### Statistical analysis

All analyses were performed using either SPSS v28 for Mac or GraphPad Prism v9.3.1. The results presented here are based on data extracted on April 25, 2022 while the study was still ongoing. Differences between groups were assessed by the Mann–Whitney *U* test or Chi-squared test, as appropriate. Differences in response times, including time to the deepest response and PFS, from the date of study enrolment, were assessed by the log-rank test and visualised by the Kaplan–Meier method when expedient. All tests were two-sided, and *P* values of less than 0.05 were considered statistically significant.

## Results

### Patients

Between May 29, 2018 and October 22, 2021, 80 patients were enrolled (Fig. 1). Four cases were screening failures or withdrew the informed consent before the first FLOX treatment was administered, leaving 76 patients randomly allocated between the study arms (patient and disease characteristics in Table 1). Of these, three control arm participants and one in the experimental arm had baseline radiologic reviews of >4 weeks at the commencement of study treatment and were therefore ineligible for the present analysis, which consequently comprised 72 patients who had received at least one dose of study therapy.

**Table 1:** Patient and disease characteristics.

	Control arm, *N* (%)	Experimental arm, *N* (%)
	38 (100)	38 (100)
Median age (min, max), years	65 (38, 79)	61 (43, 80)
Median BMI (min, max), kg/m^2^	25 (18, 40)	26 (18, 43)
Female	15 (39.5)	20 (52.6)
ECOG performance status 0	21 (55.3)	23 (60.5)
*RAS/BRAF*-mutant tumour	29 (76.3)	26 (68.4)
Left-sided primary tumour	27 (71.1)	26 (68.4)
Primary tumour in situ	20 (52.6)	16 (42.1)
Liver involvement	32 (84.2)	31 (81.6)
Median metastatic sites (min, max)	2 (1, 3)	2 (1, 5)
Synchronous metastasis	35 (92.1)	30 (78.9)

*BMI* body mass index, *ECOG* Eastern Cooperative Oncology Group, *max* maximum, *min* minimum.

Two experimental-arm patients left the study a week after the first FLOX infusion—one because of cardiac asystole (which was reversed) and the other from fatal colitis—resulting in 35 valid cases for the first post-baseline response assessment. At this point, four control arm patients had also left the study after at least one dose of study medication—one found dead at home and three with other intolerable events—resulting in 31 valid cases for the first 8-week evaluation.

### Radiologic responses

**Fig. 2:**
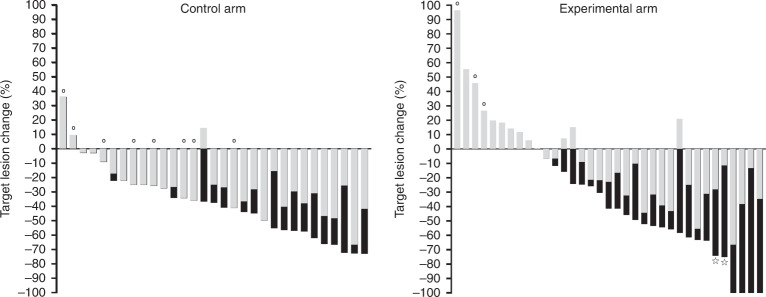
Target lesion (TL) changes for individual patients. Grey columns represent the first post-baseline response assessment, which for 14 (45.2%) control arm cases (*N* = 31) and 11 (31.4%) experimental-arm cases (*N* = 35) was the best overall response. Black columns represent the overall TL changes for the remaining cases. Symbols: circle, the patient had discontinued the study treatment before the second response assessment; star, the patient had experienced complete response of TL lymph node metastases.

At the first post-baseline response assessment (Fig. 2), the control arm patients experienced a median TL change of –27% (minimum, –67%; maximum, +36%) compared to median –11% (minimum, –67%; maximum, +96%) for the experimental-arm patients (Mann–Whitney *U*, *P* = 0.016). However, at the second response assessment, the experimental-arm patients’ TL responses had improved to the median change of –25% (minimum, –75%; maximum, +56%), not statistically different (Mann–Whitney *U*, *P* = 0.41) from the control arm patients’ median of –41% (minimum, –70%; maximum, +29%). At this point of the treatment courses, a total number of 11 (31.4%) of the initial 35 control arm patients and 5 (13.5%) of the initial 37 experimental-arm patients had discontinued study treatment due to a PFS event, intolerable toxicity or withdrawal of consent.

**Fig. 3:**
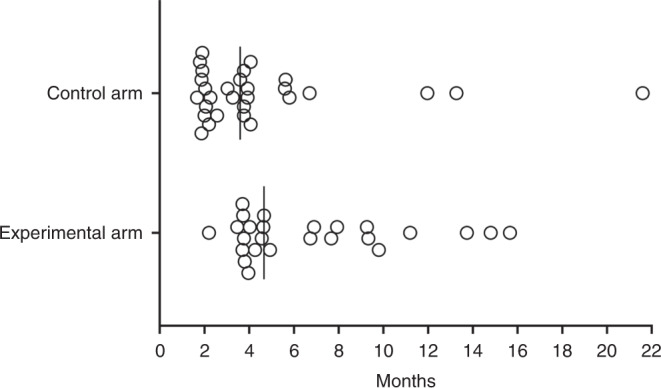
Time until the deepest target lesion reduction for the individual patients (dots) in control (*N* = 29) and experimental (*N* = 25) study arms. Vertical lines indicate the median values. Cases of only target lesion increase were excluded from this analysis.

We observed no difference (log-rank, *P* = 0.07) between the two study treatments regarding time to the first objective response (as per RECIST/iRECIST)—control arm: median of 2.1 months (95% confidence interval (CI), 2.0–2.3); experimental arm: median of 2.1 months (95% CI, 1.5–2.8). Neither did the TL objective response rates differ (Chi-squared, *P* = 0.09)—control arm: 21 of the 31 patients (67.7%) had partial response; experimental arm: 6 (17.1%) cases of complete response and 13 (37.1%) cases of partial response among the 35 patients. But the time interval until the deepest TL reduction occurred (Fig. 3) was significantly shorter (log-rank, *P* = 0.03) for control arm patients (*N* = 29) with a median of 3.6 months (95% CI, 2.8–4.4) than for experimental-arm patients (*N* = 25) with a median of 4.7 months (95% CI, 4.1–5.3). Nevertheless, the magnitude of the overall TL changes (Fig. 2) was not different (Mann–Whitney *U*, *P* = 0.65)—control arm (*N* = 31): median –37% (minimum, –73%; maximum, +36%); experimental arm (*N* = 35): median –41% (minimum, –100%; maximum, +96%).

One experimental-arm patient with a single liver TL and multiple lung non-target lesions was particularly informative with 21% TL increase at the first post-baseline assessment before a decrease to –47% of the baseline TL value at the subsequent evaluation. Because of severe myelosuppression during the second FLOX cycle, dosing of the first nivolumab cycle was 2 weeks delayed and the patient had the 8-week CT assessment 8 days after it had been administered. The patient also participated in the liver metastasis MRI programme and had acquisitions at baseline, after completion of the two initial FLOX cycles and then following the sequential two nivolumab cycles (Supplementary Fig. S2). On the third of the sequential MRI recordings, corresponding to the first post-baseline CT evaluation in patients without dosing delays, the TL change from baseline was –30%, highlighting the critical aspect of timing the response assessment to reflect the relevant treatment. Of further note over the three MRI recordings, the volume changes of the single metastasis and its ADC changes relative to the liver parenchyma (Supplementary Table S1) indicated cytotoxic elimination of tumour cells by the two FLOX cycles (volume decrease and ADC increase) before signal reversing consistent with partial recovery of cellularity after nivolumab (relative volume enlargement and ADC decline but to a higher value than at baseline).

### Prediction of the primary endpoint

**Fig. 4:**
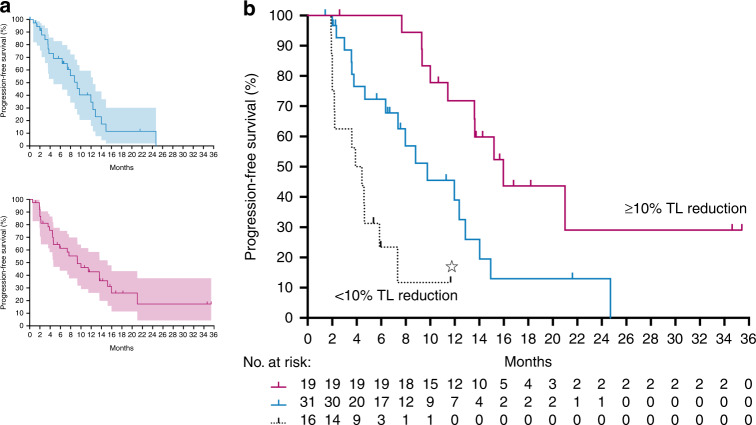
Kaplan–Meier curves for progression-free survival. Shown for all control arm patients (*N* = 35; upper panel **a**) and experimental-arm patients (*N* = 37; lower panel **a**) eligible for the present analysis, with shading reflecting the 95% confidence interval, and for the patients who reached the first post-baseline response assessment (**b**) stratified as control arm patients (*N* = 31; blue curve) and experimental-arm patients with ≥10% target lesion (TL) reduction (*N* = 19; pink curve) or <10% reduction (*N* = 16; black curve; star, the referred case participating in the magnetic resonance imaging programme of liver metastases).

At the date of data censoring for the present analysis, PFS for the cohort that started study treatment (*N* = 72; Fig. 4a) was not different (log-rank, *P* = 0.38) between the two arms—control arm (upper panel): median 8.8 months (95% CI, 5.7–11.9); experimental arm (lower panel): median 10.0 months (95% CI, 5.0–15.0). Attempting a signal of activity or failure of the highly exploratory ICB schedule at the earliest possible occasion, we identified the categorisation value of more or less than 10% TL reduction at the first radiologic restaging (Fig. 4b). The experimental-arm participants with ≥10% TL reduction at this 8-week assessment (comprising 19 of 35 cases) reached a median PFS of 16.0 months (95% CI, 12.3–19.7). In contrast, the <10% patient group (*N* = 16) had median PFS of 3.9 months (95% CI, 2.3–5.5). These outcomes were superior (log-rank, *P* < 0.01) and inferior (log-rank, *P* < 0.01) to the median PFS of 9.8 months (95% CI, 4.9–14.7) for the 31 control arm patients who reached the first post-baseline response assessment. For comparison (Supplementary Fig. S3), PFS for the ≥10% TL reduction group of experimental-arm patients remained superior to the median PFS of 12.0 months (95% CI 6.4–17.5) for the corresponding ≥10% control arm group (*N* = 25; log-rank, *P* = 0.03). Outcomes for the <10% TL reduction groups were not different with median PFS of 3.6 months (95% CI, 1.0–6.1) for the control arm (*N* = 6; log-rank, *P* = 0.71 compared with the corresponding experimental arm).

At the current censoring, 12 of the 72 patients were still under study treatment, of whom 9 were in the experimental arm. Three patients had discontinued treatment after intolerable immune-related adverse events.

## Discussion

The METIMMOX data presented here suggest that in metastatic MSS/pMMR-CRC, an early radiologic TL reduction of 10% or more can select ICB-responsive cases. However, the study entails at least three unpredictable aspects for the participants allocated to the experimental treatment arm. First, patients with unresectable metastatic MSS/pMMR-CRC, which generally is unsusceptible to ICB [11], are given ICB medication and only half the routine number of commonly efficacious chemotherapy cycles in the first-line setting. Next, the participants suffer from infradiaphragmatic metastatic disease, of whom particularly those with liver metastases may have diminished tumour-targeting immune cell function [13, 14]. Finally, the go-and-stop treatment schedule, dictated by the Nordic FLOX regimen that often compels treatment breaks to be tolerable [29], might not necessarily lead to the optimal number of repeat sequential FLOX and nivolumab cycles for the induction of a durable antitumour immune response, if occurring. Each of these reasons was an obligation for identifying an applicable and reliable signal of clinical activity or failure at the earliest possible stage of the treatment course.

The iRECIST were developed to capture both typical and atypical ICB response patterns [27]. For tumour-directed medications, assessments of durable effects on the tumour burden and PFS in randomised controlled trials are accepted as direct measures of clinical benefit while limiting patients’ and healthcare burdens. However, since iRECIST remain to be validated in prospective trials [2], we have taken a conservative approach in the METIMMOX study where chemotherapy alone is the control arm, by using RECIST version 1.1 as the primary evaluation method for all patients and iRECIST only as a subsidiary method for the experimental-arm patients, as recommended [1], at the risk of underestimating ICB responses [25, 30]. By this, the present analysis showed that tumour shrinkage was faster and initially deeper with a shorter interval until the deepest TL reduction occurred for patients receiving chemotherapy. Nevertheless, the magnitude of TL responses at the second post-baseline assessment or when at the deepest, time to the first objective response and the objective response rates were similar in the control and experimental study arms. The longitudinal tumour burden dynamics for the patients receiving the experimental treatment were in accordance with ICB responses in general [2].

Despite the slower tumour burden dynamics in the experimental study arm, the categorisation value of higher or lower than 10% TL reduction already at the first post-baseline response assessment (8 weeks) distinguished well between MSS/pMMR-CRC populations with superior or inferior PFS compared to the outcome of chemotherapy only. This early radiologic signal of clinical activity (≥10% TL reduction), if validated, may guide the selection of patients to the safe continuation of investigational ICB schedules. However, it is no guarantee against a compromised overall prognosis for patients with early ICB failure who can proceed to salvage chemotherapy. Moreover, our dataset is insufficient to suggest that the strategy would perform in identifying the metastatic MSI/dMMR-CRC subpopulation with primary ICB resistance [4]. These concerns need to be specifically addressed in prospective studies. Interestingly, prespecified radiological metrics of favourable antitumour effect at 6 weeks were recently used for adaptive dosing of a combination ICB regimen in advanced melanoma [31]. Moreover, in a retrospective cohort study of metastatic MSI/dMMR-CRC patients given ICB, the investigators observed a strong association between early tumour shrinkage in terms of TL change and an advantageous survival outcome [32], in line with our data.

In addition to clarifying the factual TL change after the four initial therapy cycles for the experimental-arm case with dosing delay and the resulting “premature” 8-week CT assessment, the findings from the patient’s exploratory liver MRI were consistent with loss of metastasis cellularity after the two FLOX cycles before partial recovery of cellularity after the sequential two nivolumab cycles. In a prospective multiparametric MRI study of ICB effects in previously untreated patients with metastatic melanoma [33], early detection of cell density loss measured by an increase in apparent diffusivity preceded tumour regression on the CT restaging. Furthermore, the investigators observed that transiently progressing lesions had diffusion changes consistent with high cell density, interpreted as tumour enlargement from immune cell infiltration rather than tumour cell proliferation. A retrospective multiparametric MRI study of ICB-treated patients with recurrent glioblastoma also found an interval increase in tumour ADC in responding patients, while ADC decrease was seen for non-responders [34]. Our single-case observations are in line with these reported data.

The present analysis is limited by a relatively low number of patients in an ongoing study with evolving results. Because of the potential hazard of the experimental-arm treatment and the resulting caution taken in the response assessment or of other safety reasons, not all cases of unconfirmed progressive disease, pursuant to the iRECIST scoring, were confirmed by consecutive imaging as required by these criteria [27]. As a consequence, cases of delayed or undulating radiologic responses under ongoing clinical activity might have been missed. However, the assessment of ≥10% or <10% TL reduction early in ICB treatment may be a simpler radiologic procedure resulting in more robust readout data and lower inter-observer variability and consequently, the opportunity of higher conformity in the endpoint determination across ICB trials, than the prevailing iRECIST scoring. The systematic comparison showed that measurement variability of ICB effects was significantly reduced with a simplified method (unidimensional versus bidimensional measurement) [35]. Of note, the 10% cut-off value was almost identical to the median value for the experimental-arm patients. Higher absolute values than 10% TL reduction caused imbalanced group sizes and, importantly, did not better predict PFS, distinguishing the 10% TL signal from objective response measures of the RECIST.

In conclusion, we identified a patient subpopulation of unresectable metastatic MSS/pMMR-CRC cases responding significantly better in the first-line setting to an investigational ICB schedule than to chemotherapy through an early occurring and simply measurable radiologic alteration, despite slowed tumour burden dynamics. This biomarker of ICB responsiveness presents itself on routine CT restaging and is a pragmatic measure for comparison with other immune-based tumour response criteria. It may ease data collection and potentially be implemented as a convenient tool to monitor exploratory ICB schedules while maintaining patient safety. The METIMMOX-2 study for patients with previously untreated metastatic MSS/pMMR-CRC (NCT05504252), building on the presented data, will start patient recruitment in September 2022. Here, ≥10% TL reduction at the first post-baseline response assessment of sequential two cycles each of FLOX and nivolumab will select patients for continuation of the study treatment. Patients who do not meet this stratification criterion will proceed to standard-of-care treatment.

## Supplementary information

Supplemental material

Consort Checklist

AJ Checklist

## Data Availability

The dataset used and analysed in this study can be made available from the corresponding author on reasonable request and in accordance with the General Data Protection Regulation of the European Union.
