# Supplementary material for: Early radiologic signal of responsiveness to immune checkpoint blockade in microsatellite-stable/mismatch repair-proficient metastatic colorectal cancer
Source: Br J Cancer. 2022 Oct 13;127(12):2227–33. doi: 10.1038/s41416-022-02004-0 (PMC9726864; doi:10.1038/s41416-022-02004-0)
Supplement: Supplementary file 1 — Supplemental material [file 41416_2022_2004_MOESM1_ESM.docx]

**SUPPLEMENTARY DATA**

**Supplementary Fig. S1**. **The METIMMOX study.** Circles indicate study visits with appendant activities. The treatment schedule consists of periods of 8 cycles. During a break period, radiologic assessment (by computed tomography; CT) and visits are done every 8 weeks until disease progression and the treatment is reintroduced in a new sequence. Adverse events are recorded at each visit. Treatment sequences are continued until disease progression on ongoing therapy, intolerable toxicity, withdrawal of consent or death, whichever occurs first.

**Supplementary Fig. S2**. **Magnetic resonance imaging of the upper abdomen of an experimental arm patient.** T2-weighted images, each with the largest cross-section of a single liver metastasis, at baseline (left panel), after completion of 2 initial chemotherapy cycles of the Nordic FLOX regimen (middle panel) and following sequential 2 nivolumab cycles (right panel).

**Supplementary Table S1.** Serial MRI readout data of the liver metastasis.

|  | **Baseline**  **MRI** | **Post-FLOX**  **MRI** | **Post-nivolumab MRI** |
| --- | --- | --- | --- |
| Volume, cubic centimetre | 7.0 | 1.8 | 4.1 |
| ADC, TL to liver parenchyma ratio | 0.81 | 0.99 | 0.89 |

Abbreviations: ADC, apparent diffusion coefficient; FLOX, the Nordic FLOX chemotherapy regimen; MRI, magnetic resonance imaging; TL, target lesion

**
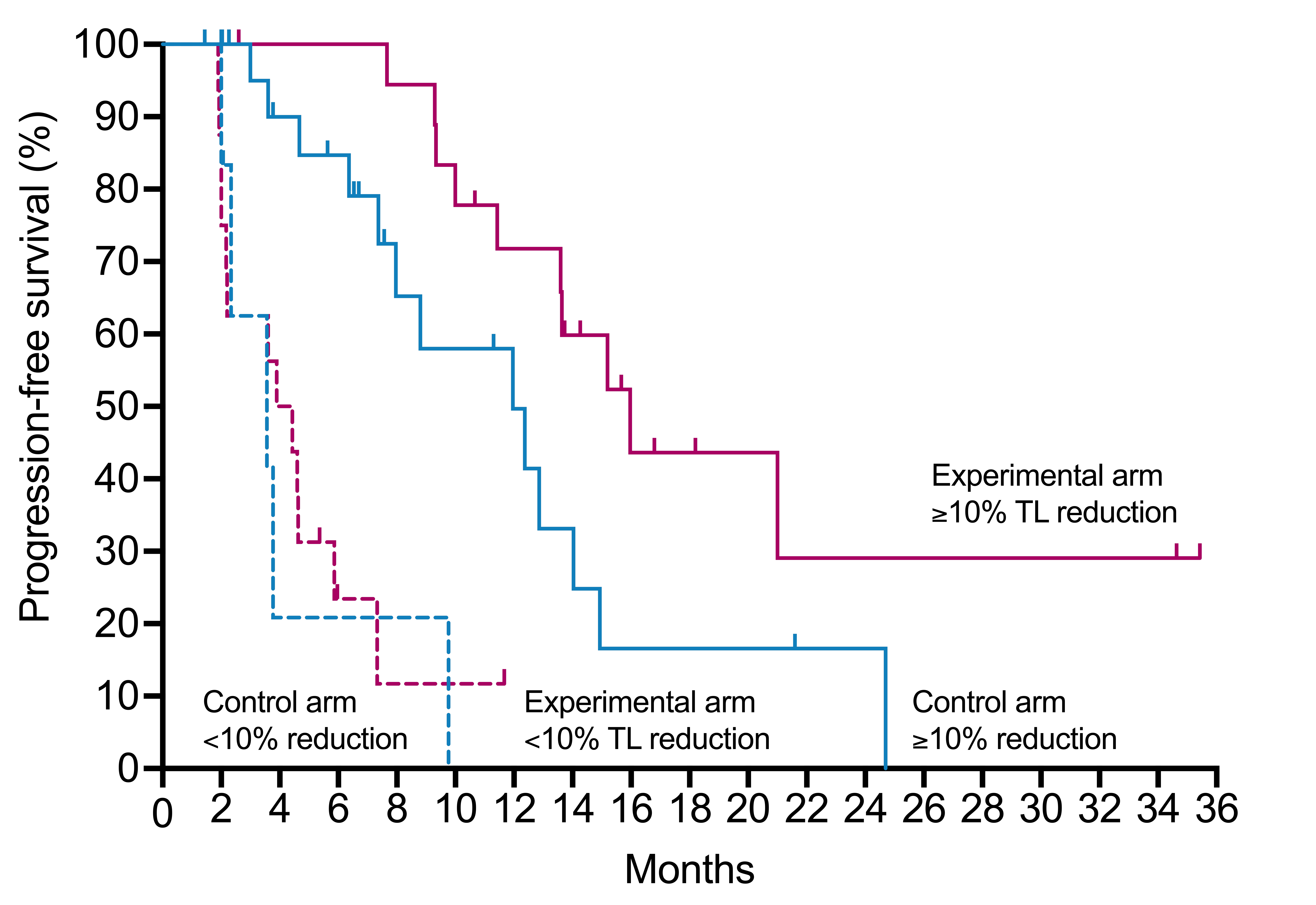
**

**Supplementary Fig. S3.** **Kaplan-Meier curves for progression-free survival.** Shown for experimental arm patients obtaining ≥10% (pink solid curve; *N* = 19) or <10% (pink broken curve; *N* = 16) target lesion (TL) reduction at the first post-baseline radiologic assessment, and the corresponding control arm patients (blue solid curve; *N* = 25 and blue broken curve; *N* = 6).
